# Carbon Paper as Current Collectors in Graphene Hydrogel Electrodes for High-Performance Supercapacitors

**DOI:** 10.3390/nano10040746

**Published:** 2020-04-14

**Authors:** Peihui Luo, Lili Huang

**Affiliations:** 1 Organic Optoelectronics Engineering Research Center of Fujian’s Universities, College of Electronics and Information Science, Fujian Jiangxia University, Fuzhou 350108, China; 2 Office of Scientific Research, Fujian Jiangxia University, Fuzhou 350108, China; 1698122945261@fjjxu.edu.cn

**Keywords:** carbon paper, graphene hydrogel, supercapacitors, current collectors

## Abstract

Current collectors are an important component of electrodes, functioning as conductive media by collecting currents from active materials and then exporting them to the external circuit. Common current collectors for graphene hydrogel (GH)-based supercapacitors are nickel foams or metal foils (platinum, gold, and aluminium, etc.). Here, hydrothermally synthesized GH was directly pressed on carbon paper and used as electrodes (denoted as GHE) for supercapacitors. With a mass loading of 2.7 mg·cm^−2^ at an active area of 0.64 cm^2^, the GHE-based supercapacitors revealed a high gravimetric capacitance of 294 F·g^−1^ at a current density of 1.18 A·g^−1^. When increasing the current density to 28.24 A·g^−1^, 66% (193 F·g^−1^) of the initial capacitance was maintained for the GHE-based supercapacitors. High performance for GHE-based supercapacitors was attributed to large specific surface area and good electrical conductivity of GH, and its intimate contact with carbon paper.

## 1. Introduction

Supercapacitors represent an important class of energy storage devices with a high power density, rapid charge/discharge rate, and long cycle life. The electrode is the core part of a supercapacitor, consisting of active materials and current collectors. The widely used active materials are transition metal compounds, conducting polymers, and carbon materials, etc. [1,2,3,4]. Besides conventional active carbon, graphene [5], especially the three-dimensional porous graphene (3DG), is an emerging material applied in the electrodes for supercapacitors among various carbon materials [6,7]. As one kind of 3DG, graphene hydrogel (GH) attracts much attention owing to its easy preparation, controllable shape, and low cost [8,9]. Synthesized GH with strong self-supporting property and good electrical conductivity can be conveniently pressed on various current collectors without additional binding agents and conductive additives. Up to now, most GH-based supercapacitors have exhibited gravimetric capacitances of 100−300 F·g^−1^ [2,6,10,11]. Nickel foams [12,13,14,15,16], platinum [17], gold [18,19], and aluminum [20] foils are usually used as current collectors for GH-based supercapacitors. Traditionally, GH-based electrodes were fabricated via mechanically or artificially compressing GH onto the flat surface of diverse metal current collectors. For increasing the contact between GH and current collector, GH was directly grown in the macropores of nickel foam to form the interconnected network structure [12]. This structure shortened the distance of ion/electron transport in the electrodes, and significantly improved its areal capacitance and rate performance for supercapacitors. In addition, in situ growth of GH film on Au foil was also achieved by electrochemical reduction strategy [21]. The prepared GH film had oriented graphene porous structure. Nickel foam with porous structure is an ideal current collector, but it needs to be cleaned with acids before use, while metal foils are hard to adhere with GH due to their glossy surfaces. Here, conductive carbon paper was prepared via thermal treatment of porous carbon fibre/carbon composites, and used as the current collectors in GH electrodes for supercapacitors. In comparison to metal foils, the carbon paper with high porosity (78%) and similar carbon component was easier to contact with GH [22,23]. Along with high conductivity (1.78 × 10^4^ S·m^−1^) and chemical stability of carbon paper, GH electrodes on carbon paper (denoted as GHE) exhibited excellent performance for supercapacitors. At the current density of 1.18 A·g^−1^, a high gravimetric capacitance of 294 F·g^−1^ was obtained and the capacitance remained 66% (193 F·g^−1^) even when the current density was elevated to 28.24 A·g^−1^. Additionally, the influences of loading mass and area of GH on carbon paper on the performance of GHE-based supercapacitors were also systematically studied.

## 2. Materials and Methods

### 2.1. Preparation of GHE

Firstly, GH was synthesized by hydrothermal treatment of graphene oxide (GO) [24,25]. 14 mL GO aqueous dispersion (2 mg·mL^−1^, Nanjing XFNANO Materials Tech. Co., Ltd., Jiangsu, China) was added into a 22 mL Telfon-lined autoclave, and hydrothermally treated at 180 °C for 4 h. The prepared cylindrical GH was picked out and kept in distilled water for further use. For fabricating GH based electrodes, GH was cut into small slices and pressed onto the hydrophilic carbon paper (TGP-H-090, Toray Industries Inc., Tokyo, Japan) with the size of ca. 1 cm × 3 cm. The mass for each dried GH was measured to ca. 12 mg. The number of the used GH slices depended on the mass of GH loading on carbon paper. GHE with mass loadings of 2.7 mg·cm^−2^ at 0.64 cm^2^, 4.1 mg·cm^−2^ at 0.68 cm^2^, and 2.7 mg·cm^−2^ at 1.07 cm^2^, were referred to as GHE1, 2 and 3, respectively. The obtained GHE were immersed in 1 M KOH aqueous electrolyte overnight before carrying out electrochemical measurements.

### 2.2. Electrochemical Measurement

The electrochemical performance of GHE-based supercapacitors was measured in 1 M KOH aqueous electrolyte. A three-electrode system was used in which GHE, Pt wire, and saturated calomel electrode (SCE) were used as work, counter, and reference electrodes, respectively. Each GHE electrode had an active area of 0.64–1.07 cm^2^ and a mass loading of 2.7–4.1 mg·cm^−2^. The data from galvanostatic charge/discharge (GCD) curves were used to calculate gravimetric capacitances according to the following formula [24]:

(1) $$C=\frac{I\cdot \Delta t}{m\left(\Delta V-IR\right)}$$

where C is the gravimetric capacitance for GHE of unit F g^−1^, I is the current applied on the GHE of unit A, Δt is the discharging time of unit s, *m* is the dried mass of GH loading on each carbon paper of unit g, ΔV is the potential difference of GCD process with unit V, and IR represents the voltage drop at the beginning of the discharge process with unit V.

### 2.3. Characterization

The interior GH morphology was observed by scanning electron microscopy (SEM, Inspect F50, FEI, Hillsboro, USA). The crystalline structure was acquired with transmission electron microscopy (TEM, Tecnai G^2^ F30, FEI, Hillsboro, USA) and X-ray diffractometer (Ultima IV, Rigaku, Tokyo, Japan), respectively. The microstructure and element analysis were recorded on a Raman spectrometer (LabRAM HR Evolution, HORIBA, Kyoto, Japan) with a 514 nm laser beam and X-ray photoelectron spectrometer (ESCALAB 250 Xi, Thermo Fisher Scientific, Waltham, USA) with Al Kα as the X-ray source and a pass energy of 30 eV, respectively. The specific surface area of Brunauer–Emmett–Teller (BET) and pore distribution analysis of Barret–Joner–Halenda (BJH) were conducted at 77 K using a gas adsorption surface area and pore size analyzer (QUADRASORB evo^TM^, Quantachrome Instruments, Boynton Beach, USA). The electrochemical properties were measured on an electrochemical workstation (660E, CHI, Shanghai, China).

## 3. Results and Discussion

**Figure 1:**
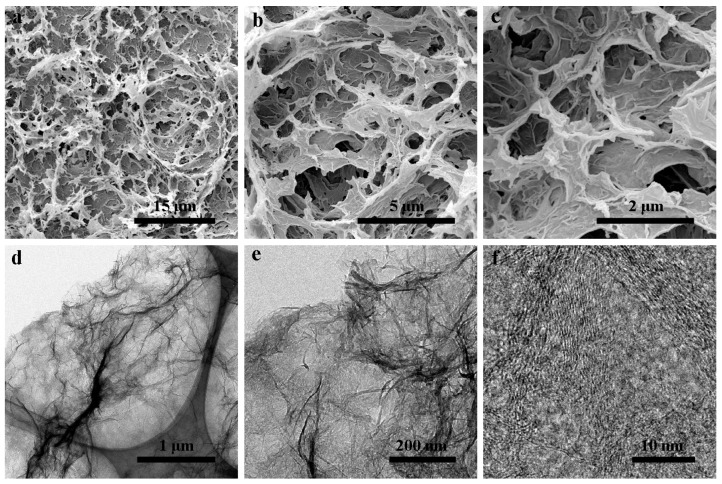
Different magnifications SEM (**a**–**c**) and TEM (**d**–**f**) images of GH morphology.

**Figure 2:**
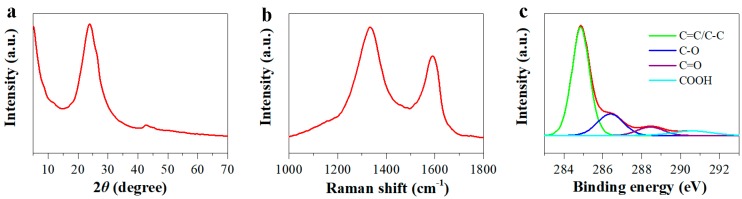
XRD (**a**), Raman (**b**) and C 1s XPS (**c**) patterns of GH.

The morphology for hydrothermally synthesized GH after freeze-drying was characterized by SEM and TEM. SEM observations clearly showed porous structure with micro-grade sizes for GH (Figure 1a–c). The wrinkle and graphite crystalline of graphene sheets from GH were seen via TEM images (Figure 1d–f). The structural and element analysis results for GH were shown in Figure 2. A X-ray diffraction (XRD) pattern revealed a typical peak at 2θ = 23.9° for the reduced graphene oxide (RGO), corresponding to the (002) peak of graphite (Figure 2a) [25]. Two obvious Raman peaks at 1333 and 1590 cm^−1^ were also presented for GH, related to D and G bands of graphite, respectively (Figure 2b). In addition, the calculated I_D_/I_G_ of 1.36 was rational for GH. X-ray photoelectron spectroscopy (XPS) analysis indicated that GH possessed a high C/O ratio of 6.5 after hydrothermal reduction. A few oxygenated groups remained for GH, including C–O (286.4 eV), C=O (288.5 eV) and COOH (290.6 eV), respectively (Figure 2c). Residual oxygenated groups benefited from the contact between GH and aqueous electrolyte, and provided partial pseudo-capacitance for GH electrodes [26,27]. These results confirm the successful preparation for GH consisting of RGO sheets.

**Figure 3:**
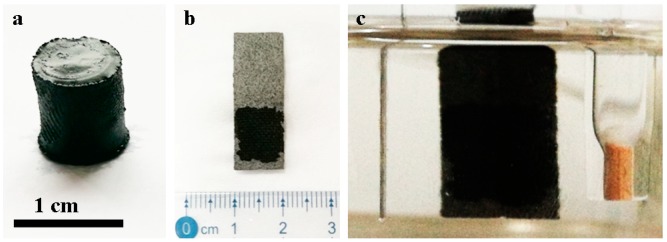
Photographs of GH (**a**), GHE (**b**), and a three-electrode system (**c**) with GHE as the work electrode.

The GH was firstly synthesized via hydrothermal reduction of GO. It had a regular cylinder shape with a diameter of ca. 0.8 cm, as shown in Figure 3a. To prepare GH-based electrodes, the synthesized GH was cut into small slices, and pressed on carbon paper evenly. A photograph for the obtained GHE was shown in Figure 3b. The GH was tightly attached to the carbon paper owing to its high porosity and similar element component with GH, while the GH slice was prone to separate from Pt foil. Before measuring supercapacitor performance, the GHE was immersed in aqueous electrolyte overnight for protecting the intrinsic porous structure and exchanging electrolyte into its interior. For performing the electrochemical measurements, GHE, platinum wire, and SCE were assembled into a three-electrode system and used as work, counter and reference electrodes, respectively, as shown in Figure 3c. Here, 1 M KOH aqueous solution was used as the electrolyte.

**Figure 4:**
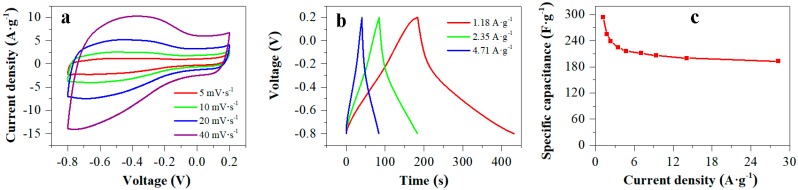
CV plots at various scan rates (**a**), GCD curves at different charging/discharging current densities (**b**) and specific capacitances versus current densities (**c**) for a GHE1-based supercapacitor.

**Figure 5:**
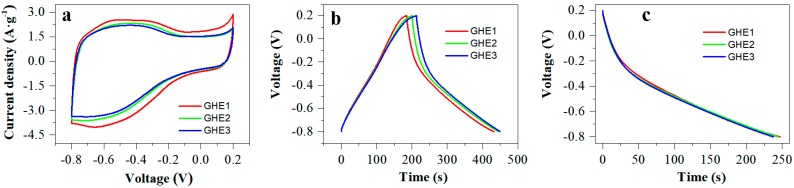
CV plots (**a**), GCD curves (**b**) and galvanostatic discharge (GD) curves (**c**) for supercapacitors based on GHE1, GHE2, and GHE3, respectively.

The GHE1 with an active area of 0.64 cm^2^ and a mass loading of 2.7 mg·cm^−2^ revealed excellent electrochemical performance for supercapacitors, as shown in Figure 4. From cyclic voltammetry (CV) curves at different scan rates, the GHE1 exhibited quasi-rectangular shape with implicit redox waves below −0.2 V, indicating the main electrical-double-layer capacitive characters (Figure 4a) [28]. A small amount of pseudo-capacitance was originated from the residual oxygenated groups of GH [18]. As the scan rates increased, the shape for CV curves was nearly unchanged, indicating GHE1 had excellent rate performance for supercapacitors. The gravimetric capacitances were calculated according to the GCD curves (Figure 4b). At a current density of 1.18 A·g^−1^, the GHE1-based supercapacitors exhibited a high specific capacitance of 294 F·g^−1^. It was improved by 48%, compared with our reported value (198 F·g^−1^ at 1.05 A·g^−1^) for a model supercapacitor using similar GH-pressed platinum foils as electrodes [24]. The gravimetric capacitance of 294 F·g^−1^ at 1.18 A·g^−1^ for GHE1 is higher than that of the most reports using metal materials as current collectors in GH-based electrodes, as shown in Table 1. The specific capacitances versus current densities were plotted in Figure 4c. When the current densities varying from 1.18 to 28.24 A·g^−1^, the capacitance for GHE1 still kept 66% (193 F·g^−1^) of the initial value. However, similar GH pressed on platinum foil only kept ca. 16% (31 F·g^−1^) of the initial capacitance (198 F·g^−1^) with the current densities increasing to 20 folds [24]. The effect of mass and area of GH loading on carbon paper on its supercapacitor performance was also studied, as shown in Figure 5. The GHE2 with an active area of 0.68 cm^2^ and a mass loading of 4.1 mg·cm^−2^ showed smaller CV area (Figure 5a) and shorter discharge time (Figure 5b,c) than GHE1 due to the higher mass loading at similar active area. As a result, the GHE2-based supercapacitors revealed a specific capacitance of 269 F·g^−1^ at 1.07 A·g^−1^, lower than that of GHE1 at 1.18 A·g^−1^. If only the active area was increased, the GHE also appeared a lower specific capacitance. For example, the GHE3 with an active area of 1.07 cm^2^ and a mass loading of 2.7 mg·cm^−2^ had a lower specific capacitance of 250 F·g^−1^ at 1.03 A·g^−1^ than that of GHE1 at 1.18 A·g^−1^. Therefore, increasing either the active area or mass loading for GH on carbon paper will weaken the performance for GHE-based supercapacitors. It is necessary to overcome this for real applications.

**Figure 6:**
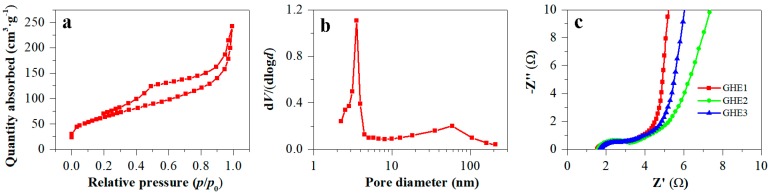
N_2_ adsorption-desorption isotherm (**a**) and the corresponding BJH pore distribution plot (**b**) of GH, and Nyquist plots (**c**) for GHE-based supercapacitors.

To get insight into the in-depth reasons for the excellent performance of GHE-based supercapacitors, further characterization was carried out. Typical IV isotherm character with a distinct hysteresis loop was presented in Figure 6a, suggesting the existence of the mesoporous structure for GH. BJH pore distribution plot for GH was shown in Figure 6b. It was seen that the main sizes for mesopores were ca. 3.5 nm. In addition, the large pores with sizes of ca. 60 nm (Figure 6b) and micro-grade (observations from SEM) also widely existed for GH. The hierarchical porous structure for GH facilitated its interior electrolyte transport. The BET specific surface area was calculated to be 228 m^2^·g^−1^ for freeze-dried GH. For more accurately evaluating the specific surface area of GH applied in the electrodes, the methylene blue (MB) adsorption method should be used to determine solvated GH samples. It can detect large pore sizes and avoid slight volume shrinkage of freeze-dried GH. For example, Zhang et al. reported hydrothermally synthesized GH with a BET specific surface area of 166 m^2^·g^−1^, while a much larger specific surface area of 964 m^2^·g^−1^ was obtained via using an MB adsorption technique [17]. Large specific surface area and good electrical conductivity from the GH itself contributed to high performance for the GHE-based supercapacitor. In addition, intimate contact GH with carbon paper further enhanced the current collection and exportation to the external circuit. Low electric resistance for the GHE was confirmed by Nyquist plots with a frequency range of 0.01 to 10^5^ Hz (Figure 6c). The diameter of the semicircle arc in the high-frequency region was lower than 2 Ω for the GHE. The straight line at the low-frequency region indicated a pure capacitive behavior. The GHE1 showed a more vertical line than that for GHE2 and GHE3, indicating more ideal capacitive behavior for the GHE1-based supercapacitors, consistent with the optimal specific capacitance of GHE1. Residual oxygenated groups located at GH surface and hydrophilicity of carbon paper also benefited from improving electrolyte transport for GHE, and enhancing their supercapacitor performance.

## 4. Conclusions

The carbon paper was used as the current collectors for the GH-based supercapacitors. With the perfect contact between GH and carbon paper, intrinsic high specific surface, and good electric conductivity for GH, the GHE-based supercapacitors exhibited excellent performance. At a current density of 1.18 A·g^−1^, the specific capacitance of GHE was up to 294 F·g^−1^, and the capacitance retention reached 66%. Even the current densities increased by ca. 24 fold. These results indicate that the GHE-based supercapacitors have high specific capacitances and excellent rate performance, and possess broad application prospects. However, increasing either mass loading or the active area of GH on the carbon paper resulted in the attenuation of the specific capacitances for GHE. Further devotion was needed to maintain outstanding supercapacitor performance for GHE with high mass loading and large active area.

## Figures and Tables

**Table 1:** Comparison of the gravimetric capacitance for GH-based electrodes on different current collectors.

Samples	Capacitance	Current Collectors	Electrolyte	References
GH	160 F·g^−^^1^ at 1 A·g^−^^1^	Platinum foil	5 M KOH	[25]
GH	222 F·g^−^^1^ at 1 A·g^−^^1^	Platinum foil	5 M KOH	[17]
Holey GH	310 F·g^−^^1^ at 1 A·g^−^^1^	Platinum or aluminum foils	6 M KOH	[29]
GH	136 F·g^−^^1^ at 1.25 A·g^−^^1^	Gold foil	1 M H_2_SO_4_	[30]
Hydroxyl-rich GH	260 F·g^−^^1^ at 1 A·g^−^^1^	Gold foil	1 M H_2_SO_4_	[18]
GH	Ca. 180 F·g^−^^1^ at 1 A·g^−^^1^	Nickel foam	6 M KOH	[31]
GH	294 F·g^−^^1^ at 1.18 A·g^−^^1^	Carbon paper	1 M KOH	This work
